# The kisspeptin analog C6 is a possible alternative to PMSG (pregnant mare serum gonadotropin) for triggering synchronized and fertile ovulations in the Alpine goat

**DOI:** 10.1371/journal.pone.0214424

**Published:** 2019-03-28

**Authors:** Caroline Decourt, Vincent Robert, Didier Lomet, Karine Anger, Marion Georgelin, Kevin Poissenot, Maria-Teresa Pellicer-Rubio, Vincent Aucagne, Massimiliano Beltramo

**Affiliations:** 1 UMR Physiologie de la Reproduction et des Comportements (INRA, UMR85; CNRS, UMR7247, Université de Tours, IFCE) Nouzilly, France; 2 Centre de Biophysique Moléculaire (CNRS UPR 4301), France; CNRS, University of Strasbourg, FRANCE

## Abstract

In temperate regions goat’s reproduction is seasonal. To obtain year-round breeding, hormonal treatments are currently applied. These treatments usually combine a progesterone analog with the pregnant mare serum gonadotropin (PMSG). However, their use has significant ethical and environmental drawbacks. Therefore, alternative methods to manage reproduction are needed. The discovery that in mammals the neuropeptide kisspeptin is a major positive regulator of hypothalamo-pituitary gonadal axis offered an attractive alternative strategy to control reproduction. We have previously designed a kisspeptin analog, called C6, which offers pharmacological advantages over endogenous kisspeptin. These include a longer lasting effect and enhanced activity following intramuscular injection. In the present work, we evaluated C6 effect on LH and FSH plasma concentrations in the Alpine goat breed and tested whether C6 could replace PMSG to trigger ovulation. An intramuscular injection of C6 (15 nmol/doe) given 24 hours after the end of progestogen treatment induced a surge-like peak of both LH and FSH. This was followed by an increase of progesterone, a hallmark of ovulation induction and *corpus luteus* formation. These results were obtained at three different time of the year: during the breeding season, the non-breeding season and at the onset of the breeding season. Furthermore, we compared the efficacy of C6 and PMSG to induce fertile ovulations when these treatments are given at the onset of the breeding season and are followed by artificial insemination. The results of this first attempt were extremely promising with gestation rates of 45% and 64% for C6 and PMSG respectively. Pending optimization of the treatment procedure in order to improve efficacy, kisspeptin analogs could be the long sought-after alternative to PMSG.

## Introduction

In temperate regions breeding seasonality of small ruminants translates into annual fluctuations of production affecting farmers’ profits. Nowadays, fertility outside the natural breeding season is achieved mainly by hormonal treatments. These are based on progestogens and pregnant mare serum gonadotropin (PMSG also called eCG for equine chorionic gonadotropin). However, the use of hormones, especially PMSG, is facing several problems. These include: reduced efficacy over time due to the production of antibody neutralizing PMSG [1], potential sanitary risks due to its extraction from animal fluid, and an increasing public hostility due to serious concerns related to animal welfare (UE report: PE 589.295v02-00 A8-0014/2017, point 43). Alternative methods, such as the so-called natural methods (which include the male effect and photoperiodic treatments), exist [2]. Nevertheless, their performance is still suboptimal and sometime compliance in the field is difficult requiring specific setting of the farming environment. Hence, new strategies to manage livestock reproduction are in demand. The discovery that neurons expressing the neuropeptide kisspeptin (Kp) are the major brain system controlling reproduction [3] has been a breakthrough and offers alternative ways to address this problem by using synthetic molecules.

Experimental evidence in mammals indicates that Kp plays a pivotal role in the stimulation of the hypothalamo-pituitary gonadal axis [4, 5]. In the ewe, intravenous infusion of Kp induced an LH surge and ovulation in both the breeding and non-breeding seasons [6]. These results prompted us to design Kp analogs having longer half-life that Kp and being active by intramuscular injection, hence more suitable than endogenous Kp for application in livestock management [7]. In the ewe one of these analogs, C6, was used to replace PMSG in a classical protocol to induce ovulation. The use of this new protocol during the breeding and non-breeding season elicited estrus behavior allowing ram servicing and good oocyte quality as demonstrated by gestation and delivery of healthy lambs at term [8].

A few studies on the goat reproductive axis were performed with either Kp or one of its analogs, TAK-683 [9]. According to the goat breed, administration of Kp was reported to be either stimulatory [10] or to have no effect on LH secretion [11]. In Shiba does, a non-seasonal breed, intravenous injection of TAK-683 applied 12 hours after removal of a CIDR (controlled internal drug-releasing device impregnated with progesterone) induced a surge-like release of LH [12]. Furthermore, TAK-683 increased LH and FSH concentrations when injected during the follicular phase induced by PGF_2_ treatment 10 days after ovulation. However, TAK-683 injection unexpectedly led to the regression of ovarian follicles rather than their maturation and ovulation[13]. These observations suggest the possibility of species-specific modulation, or organization, of the Kp in the doe and in the ewe. Alternatively, discrepancy between results obtained with TAK-683 in the doe and with C6 in the ewe could be related to the experimental setting or be drug-based.

The present study had three goals. First to establish whether C6 injection in the doe would elicit a hormonal response similar to that observed in the ewe. Second, to assess if response to C6 would depend on the breeding status of the animal (breeding vs non-breeding season vs onset of the breeding season). Third to evaluate fertility in the doe after C6 treatment and compare it to that obtained by PMSG. Taken together, our data demonstrate that in the doe C6 increases LH and FSH regardless to the breeding status and triggers fertile ovulations. We conclude that C6 might be used as an alternative to PMSG in the control of reproduction in female goats.

## Material and methods

### Animals and drugs

Experiments were conducted on Alpine does (2–8 years old) during the breeding season (January), the non-breeding (May) season, and at the onset of the breeding season (October). All animals have given birth at least once and had dried up for at least 6 months before entering the experimental protocol. Does were maintained under normal husbandry conditions at the INRA research station, (Nouzilly, Unité Expérimentale PAO n°1297 (EU0028), France) under natural daylight. Does had access to water and hay available *at libitum*, and an integration of 300 gr per day per doe of pellets containing wheat, cattle cake, lucerne, maize, oats, sugar can syrup, salt, vitamins and oligoelements. All experimental procedures were performed in accordance with international (directive 2010/63/UE) and national legislation (décret number 2013–118) governing the ethical use of animals in research. All procedures were approved by a local ethics committee (Comité d'Ethique en Expérimentation Animale Val de Loire) and an authorization was delivered by the French Ministry for Research (authorization number: Apafis1697). At the end of the experiments animals were kept alive for use in further studies.

The kisspeptin-10 analog C6 (palm-γ-Glutamyl-Tyr-Asn-Trp-Asn-Ser-GlyΨ[Tz]Leu-Arg(Me)-Tyr-NH_2_) was synthetized in house (CBM, Orléans, France) (WO2014/118318).

### Experimental design

An artificial luteal phase was induced by a classical protocol. Does received an intravaginal sponge containing a progesterone analog (Fluogeston Acetate (FA), 20 mg, Chronogest, Intervet), for 11 days. Forty-height hours before FA withdrawal does received an intramuscular injection of 125 μg of cloprostenol (0.5 mL of Estrumate, MSD) a prostaglandin F2α analog.

#### Experiment 1

The aim of this experiment was to evaluate C6 effect on LH, FSH and progesterone plasma concentrations under different reproductive statuses: during the breeding season (January), during the non-breeding season (May), or at the onset of the breeding season (October) [14]. During each one of the 3 reproductive phases a group of does (N = 10–14) was treated as described above to induce an artificial luteal phase. Twenty-four hours after FA withdrawal does received either an intramuscular bolus injection of C6 (15 nmol/doe diluted in 0.5 mL of saline, N = 5–7) or saline (0.5 mL, N = 5–7). The dose of C6 was selected based on previous data obtained in the ewe (8).

To evaluate LH and FSH plasma concentrations serial blood samples (2 mL each) were collected every 30 minutes starting 1 hour before injection and continuing up to 6 hours after injection. Then blood was sampled every hour from up to 12 hours post-injection, every 2 hours up to 18 hours post-injection, and every 4 hours up to 26 hours after injection. To assess progesterone level blood samples (2 mL) were collected daily during a week days starting from the day after C6 treatment.

#### Experiment 2

Comparison of pregnancy rates during the onset of the breeding season after C6 or PMSG treatment.

At the onset of the breeding season (October) two groups of does (N = 11 per group) were treated as described above to induce an artificial luteal phase. Does received either an im bolus of C6 (15 nmol/doe diluted in 0.5 mL of saline) 24 hours after FA withdrawal or an im bolus of PMSG (400 UI) immediately after FA withdrawal. Based on published data, does were inseminated 44 hours after FA withdrawal with frozen semen using the same ejaculate of one buck (buck number 23186720038, Capgenes, Mignaloux-Beauvoir, France). Artificial inseminations (AI) were performed by the Cooperative of Livestock Genetics and Organization (Genoe, Blain, France). Animals were than brought back to their pen until delivery.

### Hormonal assays

Plasma LH concentrations were measured using previously described radioimmunoassays [15, 16]. The assay standard was 1055-CY-LH (equivalent to 2.2 NIH-LH-S1). Intra-assay and inter-assay coefficients of variation at 2.3 ng·mL^-1^ were 5% and 9% respectively, assay sensitivity was 0.2 ng·mL^-1^.

Plasma FSH levels were measured using the reagents supplied by the NIH. The intra-assay and inter-assay coefficients of variation at 1.2 ng·mL^-1^ were 6% and 11% respectively, assay sensitivity was 0.1 ng·mL^-1^.

Progesterone was measured by an ELISA assay as previously described [8].

### Data presentation and statistical analysis

LH and FSH surges were defined as an increase in plasmatic concentration of the hormone of twice the baseline level, calculated on the median concentrations of the three samples taken before injection, in at least 2 consecutive samples. In addition, plasma concentration must have exceeded 10 ng·mL^-1^ 1 ng·mL^-1^ in at least one sample or for LH and FSH respectively. To calculate the onset and the end of the peak the first and the last values fitting the above definition were used. Data failed to pass the normality test (Shapiro-Wilk normality test) thus results are presented as the median and interquartile range or the median and 95% CI. Statistical analysis was performed by the software R3.2.2 using the permutation test. The permutation test takes into consideration interval between samples and is therefore more potent in detecting difference in samples of small sizes than a classical non-parametric test.

An increase of progesterone above the 1 ng·mL^-1^ threshold was considered as indicative of the formation of a *corpora lutea* and ovulation provided that this level was sustained until the end of the sampling period. Based on these criteria the number of ovulating animals under the different experimental conditions were compared by the Chi-square and Fischer’s exact test. Significant differences were considered when p≤0.05.

## Results

### Experiment 1. C6 treatment increases LH and FSH plasma concentrations and triggers ovulation

**Fig 1:**
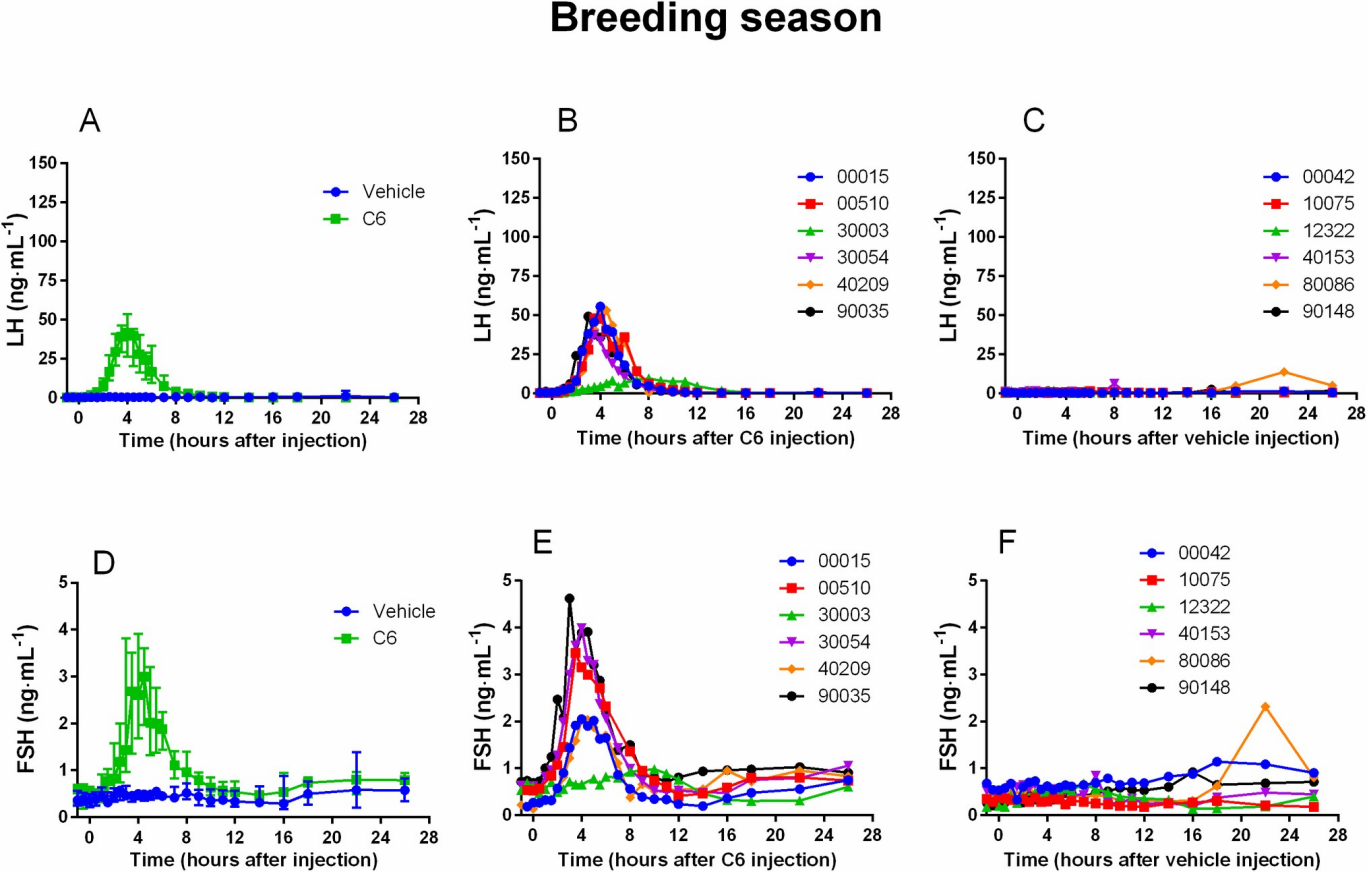
During the breeding season C6 increases LH and FSH plasma concentration. During the breeding season (January), a classical protocol of ovulation synchronization (progestogen treatment for 11 days followed by injection of prostaglandin F2α analog, cloprostenol, 48 hours before pessary removal) was applied. Two groups of does (N = 6 per group) were used. At time 0 (24 hours after pessary removal) one group received an im injection of vehicle (saline) and the other one an im injection of C6 (15 nmol/doe). The time course of LH profile following injection shows that C6 treatment induced a surge-like event (**A** median and interquartile range; **B** individual profiles) whereas in the control group there was no effect (**A** median and interquartile range; **C** individual profiles). After C6 treatment also FSH increased with a temporal profile similar to that of LH (**D** median and interquartile range; **E** individual profiles), whereas saline had no effect (**D** median and interquartile range; **F** individual profiles).

**Fig 2:**
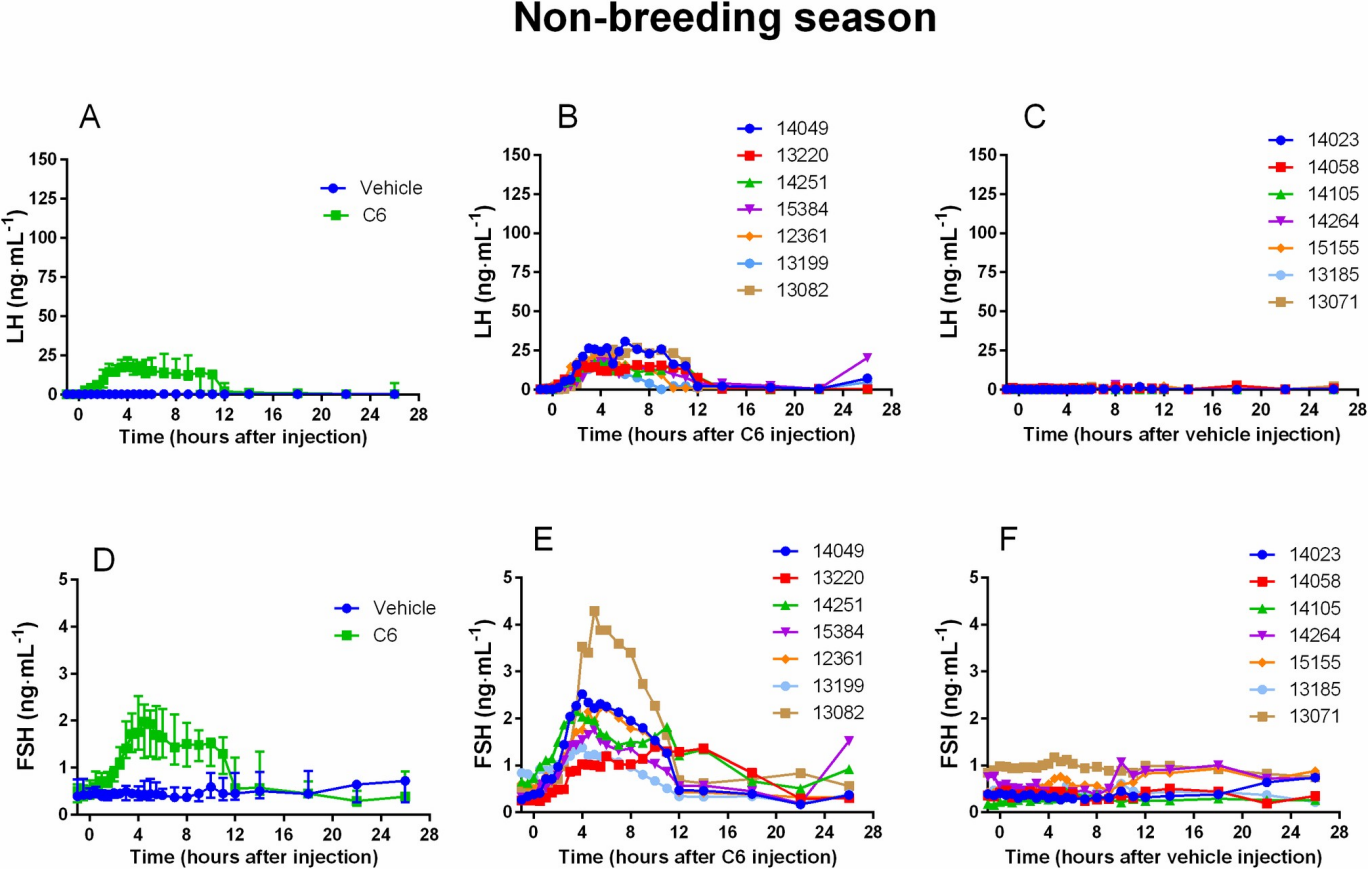
During the non-breeding season C6 increases LH and FSH plasma concentrations. During the non-breeding season (May), a classical protocol of ovulation synchronization (progestogen treatment for 11 days followed by injection of prostaglandin F2α analog, cloprostenol, 48 hours before pessary removal) was applied. Two groups of does (N = 7 per group) were used. At time 0 (24 hours after pessary removal) one group received an im injection of saline and the other one an im injection of C6 (15 nmol/doe). The time course of LH profile following injection shows that C6 treatment induced a surge-like event (**A** median and interquartile range; **B** individual profiles) whereas in the control group there was no effect (**A** median and interquartile range; **C** individual profiles). After C6 treatment also FSH increased with a temporal profile similar to that of LH (**D** median and interquartile range; **E** individual profiles), whereas saline had no effect (**D** median and interquartile range; **F** individual profiles).

**Fig 3:**
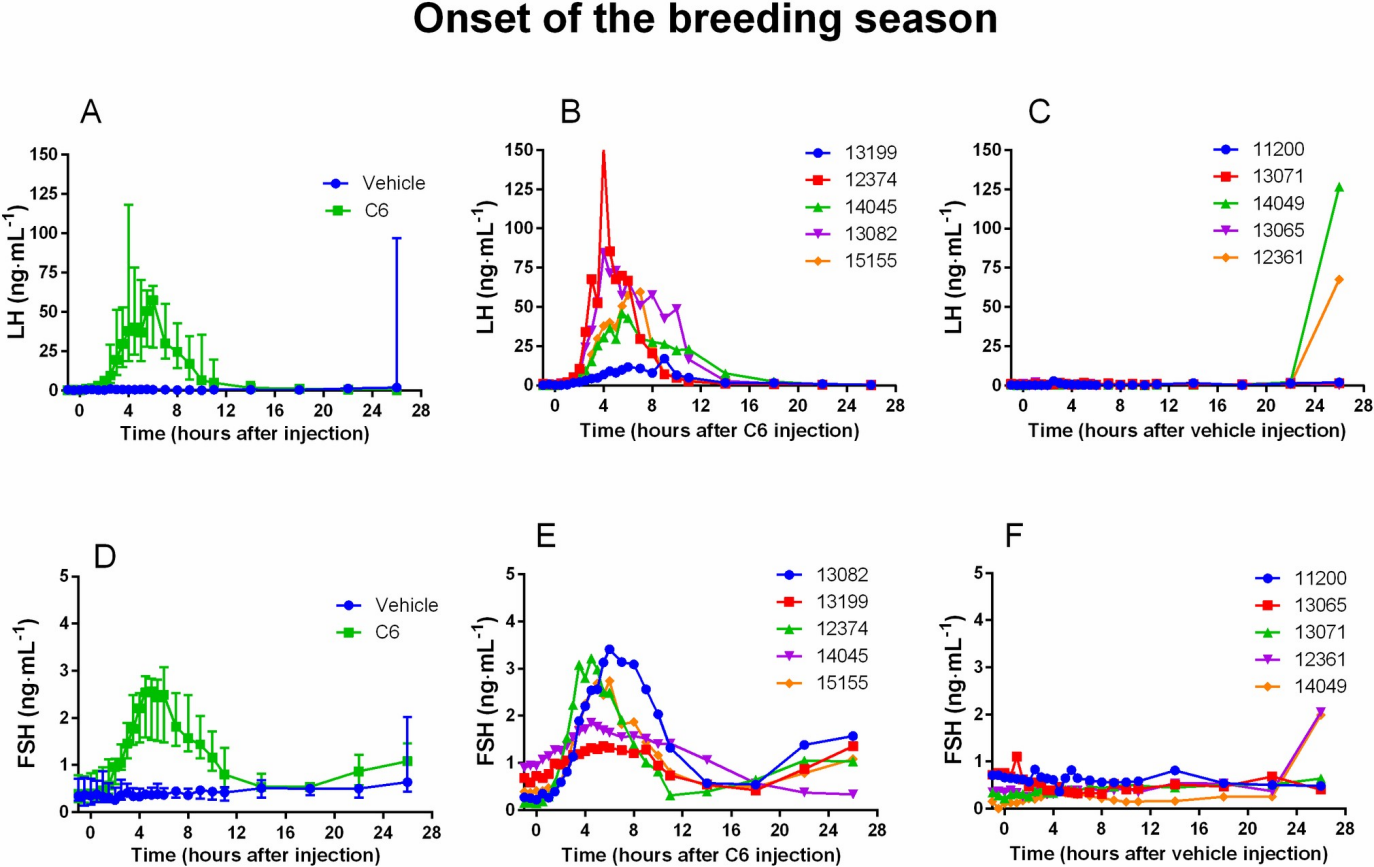
During the onset of the breeding season C6 increases LH and FSH plasma concentrations. During the onset of the breeding season (October), a classical protocol of ovulation synchronization (progestogen treatment for 11 days followed by injection of prostaglandin F2α analog, cloprostenol, 48 hours before pessary removal) was applied. Two groups of does (N = 5 per group) were used. At time 0 (24 hours after pessary removal) one group received an im injection of saline and the other one an im injection of C6 (15 nmol/doe). The time course of LH profile following injection shows that C6 treatment induced a surge-like event (**A** median and interquartile range; **B** individual profiles) whereas in the control group there was no effect (**A** median and interquartile range; **C** individual profiles). After C6 treatment also FSH increased with a temporal profile similar to that of LH (**D** median and interquartile range; **E** individual profiles), whereas saline had no effect (**D** median and interquartile range; **F** individual profiles).

Regardless of the period (breeding, non-breeding season or onset of the breeding season) C6 increased plasma LH concentration (comparison of AUC of C6-treated animals vs vehicle-treated animals P<0.001) leading to a surge-like profile in all does (panel A and B of Figs 1, 2 and 3). No interaction between season and treatment on AUC was observed (P = 0.32). Similar results were obtained with FSH (panel D and E of Figs 1, 2 and 3) (C6-treated animals vs vehicle-treated animals P<0.001, interaction season and treatment P = 0.61). Both LH and FSH surges were highly synchronous reaching their maximal amplitude at about the same time (Table 1). Conversely, saline had no effect on either LH or FSH plasma concentration (panel A, C, D and F of Figs 1, 2 and 3).

**Table 1:** Comparison of C6 effect on hormonal profile parameters during the yearly reproductive cycle.

	LH (median and 95% CI)			FSH (median and 95% CI)			Progesterone (median and 95% CI)		
	Breeding season	Non-breeding season	Onset of the breeding season	Breeding season	Non-breeding season	Onset of the breeding season	Breeding season	Non-breeding season	Onset of the breeding season
Peak onset	1 (0.5–1 hour)	0.5 (0.5–1 hour)	1 (0.5–1.5 hours)	2 (2.0–3.0 hours)	2.25 (1.0–3.0 hours)	2.25 (1.5–4.5 hours)	4 (3–5 days)	3 (2–4 days)	3 (3–4 days)
Time of peak maximum	3.7 (3.1–4.7 hours)	5 (3.5–6.4 hours)	6 (3.7–7.1 hours)	4 (3.0–4.5 hours)	5 (3.5–14.0 hours)	5.75 (4.5–6.0 hours)	_	_	_
Peak duration	10 (7–15 hours)	13.5 (8.5–25.5 hours)	20.5 (10–25 hours)	6 (5.0–24.0 hours)	9.75 (8.5–15.0 hours)	8.5 (1.5–24.0 hours)	_	_	_
AUC	159.1 (98.9–187.3 ng·h·mL^-1^)	172.7 (103.4–297.7 ng·h·mL^-1^)	312.1 (55.8–632 ng·h·mL^-1^)	12.2 (7.7–17.6 ng·h·mL^-1^)	13.6 (2.5–25.3 ng·h·mL^-1^)	17.0 (5.0–30.4 ng·h·mL^-1^)	_	_	_
Maximal amplitude	48.6 (23.4–60.4 ng·mL^-1^)	25.8 (10.7–57.4 ng·mL^-1^)^a^	59.6 (2.0–139.5 ng·mL^-1^)^a^	2.9 (1.8–3.9 ng·mL^-1^)	1.5 (0.5–3.8 ng·mL^-1^)	2.4 (0.7–3.5 ng·mL^-1^)	4.6* (0.25–12.7 ng·mL^-1^)	5.2* (4.4–6.0 ng·mL^-1^)	7.6* (4.1–11.0 ng·mL^-1^)

*Amplitude on the last sampling day. Statistical analysis was performed by applying the permutation test.

^a^ indicates a difference between onset of the breeding season and the non-breeding season (P<0.05).

The maximal amplitude reached by LH after C6 treatment was larger at the onset of the breeding season compared to the non-breeding season (P<0.05). No statistical difference was detected for the breeding season. Comparison of the AUC of the LH surge-like profile of treated animals during the three reproductive phases revealed a global difference (p<0.05; Table 1). However, the multiple comparison test found no significant difference among groups. No difference was detected for the other parameters taken into consideration (time to peak, time of peak onset, peak duration and time to maximal amplitude) for the analysis of the LH peak. No differences were found for the FSH peak. Finally, progesterone parameters did not differ between C6 treated groups (p>0.05; Table 1).

**Fig 4:**
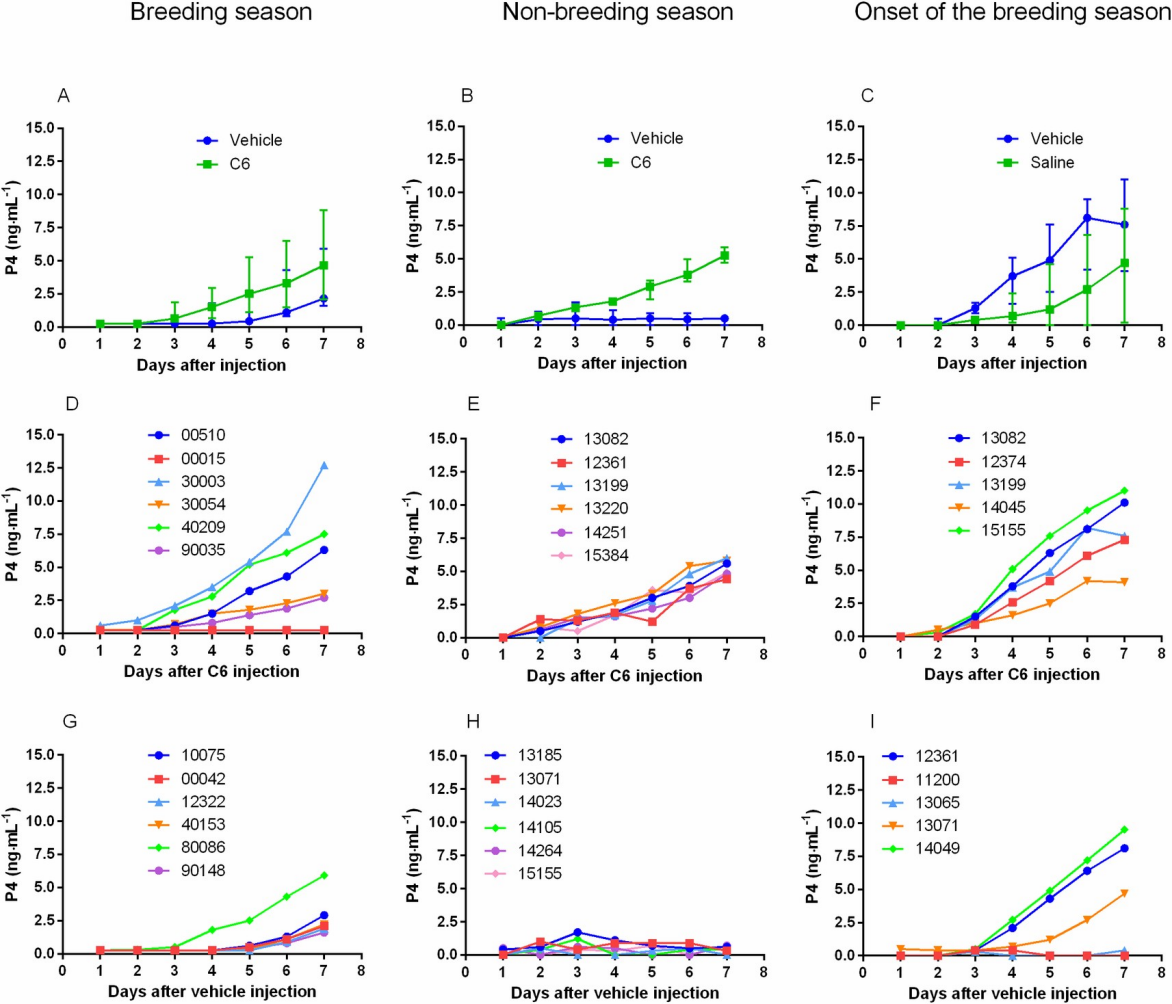
Time course of progesterone levels after C6 or vehicle treatment. A classical protocol of ovulation synchronization (progestogen treatment for 11 days followed by injection of prostaglandin F2α analog, cloprostenol, 48 hours before pessary removal) was applied to two groups of does (N = 5–6) during the breeding and non-breeding season as well at the onset of the breeding season. At time 0 (24 hours after pessary removal) one group received an im injection of saline and the other one an im injection of C6 (15 nmol/doe). Plasma progesterone level was monitored for 7 days. During the non-breeding season progesterone increased over time in all does of the treated group (**B** median and interquartile range and **E** individual profiles) but not in the vehicle treated does (**B** median and interquartile range and **H** individual profiles). During the breeding season and the onset of the breeding season progesterone increased in both C6 (**A** and **C** median and interquartile range; **D** and **F** individual profiles) and vehicle treated groups (**A** and **C** median and interquartile range; **G** and **I** individual profiles). However, after C6 treatment, progesterone increase tended to occur earlier and reached higher levels than in vehicle treated groups.

During the non-breeding season, none of the vehicle-treated animals showed an increase of progesterone level following treatment (Fig 4B and 4H) while, progesterone crossed the 1 ng-mL^-1^ threshold in all C6- treated does (Fig 4B and 4E). During the breeding season and at the onset of the breeding season most vehicle treated does showed an increase of progesterone concentration (6/6 and 3/5 does, respectively), which was not different from that observed in C6-treated animals (5/6 and 5/5 respectively) (Fig 4D and 4F for C6 treated does and G and I for vehicle treated does). Statistical analysis revealed a significant effect of the season in vehicle treated animals (P<0.05). Finally, progesterone levels tended to show a delayed increase and be lower on the 7th days in vehicle-treated animals compared to C6-treated animals (Fig 4A and 4C).

### Experiment 2. C6 treatment induces fertile ovulations

In does treated during the onset of the breeding season twenty-one days after artificial insemination (AI) plasmatic levels of progesterone were high in the two groups (7/ 11 and 8/11 does for C6 and PMSG groups, respectively; Table 2). Pregnancy rate, as established by delivery, was similar in both groups (5/11 and 7/11 for C6 and PMSG treated does, respectively; Table 2). Gestation length, prolificacy and kids’ weight were similar between the two groups (Table 2). In the C6-treated group the sex ratio was skewed, with females prevailing. Nevertheless, there was no difference compared to the PMSG group.

**Table 2:** Characteristics of the groups and results of the artificial insemination study.

	C6	PMSG
Group size	11	11
Date of AI	October 7^th^	October 7^th^
Early gestation diagnostic*	7 (64%)	8 (73%)
Confirmed pregnancy	5 (45%)	7 (64%)
Gestation length (days)	154 (152–156)	153.5 (151–156)
Prolificacy (healthy kids/doe)	1.8	1.7
Size of litters	Singleton 1, twins 3, triplets 1	Singleton 1, twins 3, quadruplets 2
Stillborn kids	1	3
Sex ratio	70% F and 30% M	47% F and 53% M
Mean weight (Kg)	4.2 (3–4.7)	3.9 (2.3–4.3)

*Established by progesterone dosing 21 days after artificial insemination. Data are expressed as median and 95% CI.

## Discussion

The kisspeptin analog C6 induced highly synchronous ovulation in does and successfully replaced PMSG treatment. We conclude that Kp analogs might be an alternative to PMSG in reproduction control. Because C6 is a synthetic molecule its use would present clear advantages over PMSG. The use of C6 would eliminate potential sanitary risks and lessen concerns about animal welfare which arise from the use of PMSG extracted from the blood of pregnant mares usually kept under poor husbandry conditions). The classical hormonal protocol to induce ovulation in small ruminants consists of a pessary containing a progestogen combined with an injection of PMSG at pessary removal. PMSG has a dual LH/FSH-like action which stimulates the ovary and promotes follicular growth and maturation. Previous studies in the ewe have shown that both Kp and C6 increase LH and FSH plasma concentrations [6, 8]. However, while Kp has to be perfused for at least 24-hours to trigger a surge-like increase of LH and FSH, a single intramuscular injection of C6 is sufficient to trigger similar surges in gonadotropins. This is due to the longer half-life and an improved resistance to degradation.

Hence, stimulation of the Kp system, leading to the secretion of LH and FSH would mimic PMSG treatment, but by acting upstream to the ovary. This hypothesis was successfully tested in the ewe both in the breeding and the non-breeding seasons [8]. Ovine and caprine are phylogenetically close relatives and show similar hormonal profiles during the ovarian cycle. We therefore predicted that C6 would produce in the goat results comparable to that obtained in the sheep. However, data on the stimulation of the Kp system in the goat, either by Kp or its analogs, are scanty and inconsistent. Most of the studies were performed in Shiba does, a non-seasonal breed. Only one study investigated the effect of Kp in a seasonal goat breed: the Abadeh breed. In Shiba does, injection of Kp10 at different doses (0.77, 3.85 or 7.69 nmol kg^-1^ of body weight) during the luteal phase of the estrous cycle triggered a rapid, but short lasting, increase of plasma LH [10]. Conversely, in the Abadeh does during seasonal anestrous Kp10 (1 μg kg^-1^ corresponding to 0.77 nmol·kg^-1^) had no effect on LH plasma level [11]. Multiple reasons could explain this discrepancy: i) the dose of Kp used in the Abadeh doe may have been insufficient to reactivate a dormant reproductive axis, ii) Abadeh and Shiba breeds may have difference sensitivity to Kp, or iii) exhibit functional differences of the Kp system. Considering the interest of managing reproduction during the non-breeding season, the last possibility would be a major drawback for the broad application of a Kp-based treatment. Our previous data in the ewe obtained in the seasonal Ile de France breed [6, 17], would tend to rule out this possibility. We decided to investigate this aspect in the Alpine goat, a highly seasonal breeder under temperate latitude. Experiments were performed at different times of the seasonal reproductive cycle (breeding season, non-breeding season, and onset of the breeding season). Regardless of the reproductive status, administration of C6 after progestogen pretreatment induced a rapid increase of LH levels. The amplitude and duration of the LH increase were similar to those reported for a natural pre-ovulatory LH surge in the doe [18, 19] and in the ewe [20, 21]. FSH also increased with a temporal profile reminiscent of a natural ovulatory cycle [22]. The maximal amplitude reached by LH plasma concentration was larger when C6 was injected at the onset of the breeding season than during the non-breeding season. A trend towards differences in the AUC was seen but failed to reach statistical significance, likely due to the small sample size and the large inter-individual variability. The lower amplitude of the LH peak during the non-breeding season might reflect reduced amounts of GnRH and/or LH available for release at this time of the year. The physiological significance of these differences in LH concentration, if any, is uncertain since no difference was found in the ovulation rate across C6-treated groups. We conclude that C6 triggers a similar hormonal response throughout the year, which culminates in the reactivation of a quiescent reproductive axis during the non-breeding season. This rule outs the hypothesis of a reduced sensitivity of the system during the seasonal anestrous.

We also verified if the gonadotropin surge would lead to ovulation and the formation of a *corpus luteum*. Available studies on does of the Shiba breed using TAK-683 yielded mixed results. Intravenous treatment with TAK-683 (35 nmol per doe) applied 12 hours after removal of a CIDR containing 300 mg of progesterone, and following PGFα2 treatment, induced a surge-like release of LH. After treatment follicles kept growing and some of them ovulated as assessed by transrectal ultrasonography [23]. The same dose of TAK-683 was tested during a follicular phase induced by PGFα_2_ treatment 10 days after ovulation. Under these conditions, however, despite the increase in LH and FSH concentrations, follicles present at the time of TAK-683 injection instead of ovulating regressed [13]. New follicles then emerged and ovulated but these were of smaller diameter and progesterone concentration after ovulation was reduced compared to control [13].

In our experimental protocol we decided to inject C6 24 hours after progestogen withdrawal. The rationale for this is that C6 injection induces a pre-ovulatory LH peak which triggers ovulation within less than 6 hours. During a natural ovulatory cycle, there is a significant delay between progesterone decline and the subsequent LH increase and ovulation. In the doe, LH peaks about 50 hours after luteal progesterone drop back to basal level [24]. This allows the follicle to complete its growth and ensure proper oocyte maturation. We reasoned that, if injected immediately after progestogen withdrawal, C6 might trigger premature ovulation of follicles and the release of incompletely mature oocytes. Therefore, C6 injection 24 hour after progestogen withdrawal appeared as a reasonable option, which would also be suitable for application in the field. Our findings validate this experimental setting as progesterone levels started to increase by day 3 after treatment and were still increasing by day 7. The *corpora lutea* generated after C6 treatment released progesterone at a levels similar to those measured during a natural cycle. Furthermore, progesterone patterns were similar for the three periods of the reproductive cycle.

As anticipated, priming with a progestogen followed by vehicle injection was sufficient to trigger ovulation during the onset of the breeding season and during the breeding season. Conversely, this treatment was inefficient during the non-breeding season; progesterone concentration remained at basal level implicating that none of the does had ovulated. Therefore, C6 is mandatory to trigger ovulation during the non-breeding season, and it is crucial to obtain highly synchronous ovulations at other times of the year.

The most compelling way to establish the quality of oocytes is to assess their capacity to yield a viable offspring. To this aim, the use of AI is preferable since it avoids confounding effects due to difference in fertility of male goats. In addition, it allows a qualitative comparison with data obtained using PMSG. Studies performed in goats by AI following classical hormonal treatments resulted in different pregnancy rates pending on protocol, breed and latitude. A precise comparison is therefore difficult but an overview would be useful to frame the results we obtained with C6 and PMSG into a more general perspective. During the breeding season CIDR treatments of variable duration (5–16 days) followed by PMSG injection (dose ranging from 100 to 300 IU) resulted in kidding or pregnancy rate of 47% in Boer breed, 63% in Nubian breed, and 50% to 93% in Saanen breed [25–27]. Using pessary containing FA, PGFα2 plus PMSG treatment, followed by AI, a 58% pregnancy rate was obtained in the Bulgarian White milk goat during the breeding season [28]. Experiments performed during the non-breeding season using either progesterone or FA followed by PGFα2 plus PMSG treatment yielded pregnancy rates of 75% and 47% in Alpine x Saanen or Murciano-Granadina does, respectively [29, 30]. Data from France indicate a global success rate of about 65% during the non-breeding season [31]. Our experimental protocol applied to Alpine does at the onset of breeding season led to a pregnancy rates of 45% and 64% for C6-treated and PMSG-treated does respectively. Remarkably, the pregnancy rate obtained with C6 is well within the range of the one obtained applying classical hormonal protocols, which flags C6 as an outstanding candidate for the management of breeding in does.

We observed a skewed sex ratio in the C6-treated group, with more females than males. However, this result should be interpreted with much caution since the number of kids is very small (n = 10). Our unpublished data on the ewe (Decourt et al.) do not support a skewed sex ratio after C6 treatment. Further experiments will be required to provide a definitive conclusion.

Finally, a further potential asset of C6 is the reduction of technical problems due to a decreased efficacy of PMSG over time. In the ewe repeated injections of C6 (up to four injections) at different time intervals from a few weeks to several months, consistently produced an LH surge (Decourt et al., unpublished data). These results suggest that, unlikely what seen for PMSG, the efficacy of C6 treatment may remain stable over time. Two main arguments may explain this difference between C6 and PMSG: i) C6 is smaller than PMSG (MW of 1724 Da vs MW range between 49000 and 68500 Da, respectively) which makes C6 less prone to trigger an immune reaction that impairs the efficacy of subsequent injections; ii) C6 maximal effect is achieved within 6 hours from injection, duration likely too short for mounting an immune response capable of preventing its effect.

In conclusion, our results demonstrate that C6 triggers fertile ovulations in the goat. However, to fully appreciate C6 potential in the management of small ruminant reproduction, large-scale direct comparisons with PMSG will have to be performed. The first step towards this goal will be to optimize the experimental protocol for C6 use. This means identifying the optimal balance between delivery time after FA withdrawal and the dose of C6 injected, in order to achieve the best pregnancy rate. Meeting this would be a breakthrough, as it would address legitimate public concern about welfare of farm animals while maintaining productivity and financial income of farmer. The next exciting challenge would be to establish whether Kp analogs can be used in breeding control of other domestic species and whether they can be applied in *ex-situ* reproductive programs of endangered wildlife species.

## Supporting information

S1 Dataset Raw experimental data. (PDF)
