# Supplementary material for: The kisspeptin analog C6 is a possible alternative to PMSG (pregnant mare serum gonadotropin) for triggering synchronized and fertile ovulations in the Alpine goat
Source: PLoS One. 2019 Mar 28;14(3):e0214424. doi: 10.1371/journal.pone.0214424 (PMC6438634; doi:10.1371/journal.pone.0214424)
Supplement: S1 Dataset — (PDF) [file pone.0214424.s001.pdf]

Breeding season LH (ng mL^{-1})

Time (hours)	Vehicle						C6					
-1	0,7	0,4	0,1	1,2	0,3	0,4	0,4	0,3	0,3	0,1	0,7	0,7
-0,5	0,4	0,3	1,4	0,6	0,2	0,3	0,4	0,6	0,2	0,4	0,3	1,2
0	0,2	0,3	0,3	0,3	0,3		0,4	0,3	0,2	0,1	0,2	0,5
0,5	0,3	0,3	0,2	0,8	0,4		1,1	0,6	0,3	0,5	0,6	1,3
1	0,2	0,4	1,7	1,3	0,4	0,2	2,2	1,4	0,5	2,2	1,3	3
1,5	0,5		0,3	1,4	0,2	0,1	3,2	4,1	1,2	3,8	2,8	6
2	0,3	0,5	0,3	0,7	1,3		7,8	8,2	1,5	8,6	5,8	24,1
2,5	0,6	0,2		0,3	1,6	1,5	26,9	17,3	2,5	16,3	13,9	27,8
3	0,4	0,7	1,8	1,0	0,4	0,3	38,1	27,9	3,1	30,6	26,4	49,2
3,5	0,3	0,3	0,6	0,5	0,9	0,1	45,7	48	4,1	37,5	41	37,6
4	0,4	0,8	0,3	0,8	0,3	0,1	55,5	47,5	4,6	33,6	52,7	35,8
4,5	0,3	0,4	0,2	1,1	0,6	0,6	40,9	40,1	6,4	24,9	53	39,3
5	0,2	1,0	0,2	0,6	0,4	0,4	39,2	30	8,2	19	43,4	25,9
5,5	0,6	0,5	0,8	0,3	1,6	0,2	24,1	27,7	4,3	14,2	32,7	24,3
6	0,3	1,3	0,3	0,3	0,4	0,1	18,1	35,8	6,8	10,3	32,7	15
7	0,4	0,9	0,2	0,2	0,7	0,2	6,3	14	8,6	6	14,6	5,6
8	0,6	0,9	0,3	6,1	0,4	0,9	4,8	6	9,5	2,9	0,5	3,4
9	0,5	0,5	0,1	0,1	0,4	0,1	2,1	3,7	8,1	1,6	3,3	1,5
10	0,2	0,5	0,3	0,5	0,8	0,7	1,2	2,1	7,5	0,9	1,7	1
11	0,3	0,3	0,2	0,2	0,5	0,2	0,7	1,2	7,5	0,6	1	0,6
12	0,3	0,3	0,3	0,4	0,4	0,4	0,4	0,6	4,6	0,4	0,7	0,3
14	0,8	0,6	0,3	0,2	0,5	0,2	0,3	0,2	1,8	0,3	0,6	0,2
16	0,3	1,0	0,1	0,2	0,9	2,4	0,3	0,2	0,5	0,1	0,2	0,1
18	1,3	0,6	0,1	0,7	4,6	0,2	0,3	0,2	0,2	0	0,2	0,1
22	1,3	0,4	0,4	1,2	13,6	0,8	0,6	0,1	0,1	0,1	0,1	0
26	0,4	0,4	0,4	0,4	4,9	1,6	0,2	0,1	0,1	0,1	0,3	0,1

Breeding season FSH (ng mL^{-1})

Time (hours)	Vehicle						C6					
-1	0,677	0,347	0,177	0,525	0,38	0,265			0,536	0,633	0,22	0,714
-0,5	0,54	0,286	0,3	0,518	0,474	0,202	0,181	0,541	0,686	0,586	0,183	0,736
0	0,53	0,374	0,195	0,471	0,354	0,225	0,27	0,529	0,611	0,619	0,132	0,688
0,5	0,579	0,355	0,181	0,516	0,408	0,272	0,263	0,569	0,474	0,566	0,256	0,721
1	0,672	0,324	0,413	0,626	0,457	0,353	0,325		0,556	0,824	0,325	1,004
1,5	0,328		0,265	0,604		0,299	0,316	0,838	0,705	0,959	0,345	1,244
2	0,604	0,346	0,409	0,642	0,497	0,269	0,573	1,069	0,494	1,272	0,49	2,468
2,5	0,697	0,284		0,574	0,592	0,436	0,904	1,452	0,551	1,975	0,809	2,075
3	0,727	0,375	0,48	0,652	0,44	0,331	1,435		0,71	2,997	1,217	4,622
3,5	0,551	0,279	0,483	0,536	0,441	0,351	1,916	3,456	0,651	3,616	1,585	3,469
4	0,617	0,293	0,501	0,5	0,497	0,409	2,05	3,157	0,656	3,989	2,024	3,89
4,5	0,539	0,362	0,541	0,55	0,439	0,33	1,902	2,997		3,293	2,035	3,911
5	0,59	0,341	0,518	0,38	0,508	0,459	2,014		0,779	3,205	1,874	3,208
5,5	0,638	0,228	0,583	0,515	0,593	0,473	1,626	2,716	0,648	2,37	1,626	2,873
6	0,616	0,308	0,522	0,439	0,416	0,465	1,649	2,321	0,825	2,059	1,704	2,211
7	0,643	0,285	0,678	0,433	0,389	0,403	0,859		0,799	1,433	1,107	1,385
8	0,687	0,246	0,562	0,844	0,426	0,473	0,561	1,361	0,924	0,986	0,377	1,496
9	0,784	0,228	0,485	0,269	0,4	0,512	0,388	0,954	0,957	0,722	0,657	0,861
10	0,648	0,195	0,399	0,247	0,308	0,58	0,343	0,738	0,983	0,533	0,514	0,808
11	0,7	0,196	0,374	0,361	0,303	0,538	0,335	0,608	0,891	0,495	0,531	0,714
12	0,686	0,173	0,357	0,245	0,315	0,528	0,235	0,409	0,754	0,529	0,551	0,807
14	0,823	0,248	0,33	0,262	0,297	0,602	0,194	0,459	0,461	0,487	0,561	0,935
16	0,875	0,269	0,136	0,193	0,31	0,92	0,362	0,593	0,328	0,482	0,946	0,959
18	1,141	0,307	0,145	0,374	0,619	0,647	0,476	0,797	0,302	0,758	0,714	0,976
22	1,084	0,212	0,187	0,481	2,312	0,683	0,555	0,803	0,311	0,789	0,951	1,026
26	0,902	0,178	0,396	0,445	0,804	0,71	0,747	0,74	0,608	1,056	0,83	0,908

Breeding season progesterone (ng mL^{-1})

Time (days)	Vehicle						C6					
1	0,25	0,25	0,25	0,25	0,25	0,25	0,25	0,25	0,6	0,3	0,25	0,25
2	0,25	0,25	0,25	0,25	0,3	0,25	0,25	0,25	1	0,25	0,25	0,25
3	0,25	0,25	0,25	0,25	0,5	0,25	0,25	0,6	2,1	0,7	1,8	0,5
4	0,25	0,25	0,25	0,25	1,8	0,25	0,25	1,5	3,5	1,5	2,8	0,8
5	0,5	0,6	0,25	0,3	2,5	0,4	0,25	3,2	5,4	1,8	5,2	1,4
6	1,1	1,3	0,9	1,1	4,3	0,8	0,25	4,3	7,7	2,3	6,1	1,9
7	2,1	2,9	1,9	2,2	5,9	1,6	0,25	6,3	12,7	3	7,5	2,7

Non-breeding season LH (ng mL^{-1})

Time (hours)	Vehicle							C6						
-1	0,34	0,35	0,06	0,14	0,17	0,07	0,25	0,19	0,21	0,1	0,14	0,12	0,03	0,15
-0,5	0,34	1,23	0,04	0,11	0,17	0,03	0,22	0,18	0,5	0,08	0,13	1,04	1,12	0,13
0	0,24	0,59	0,06	0,12	0,17	0,05	0,28	0,22	0,88	0,12	0,14	2,36	1,95	0,11
0,5	0,14	0,45	0,02	0,11	0,16	0,03	0,21	1,29	3,3	2,82	1,21	2,77	2,61	0,14
1	0,12	0,4	0,05	0,11	0,18	0,21	0,2	5,28	6,49	4,45	1,41	6,02	4,42	0,46
1,5	0,07	0,42	0,07	0,18	0,18	0,08	0,17	6,26	6,24	5,73	2,35	14,64	8,21	2,03
2	0,08	0,82	0,1	1,21	0,15	0,06	0,15	15,81	11,37	8,11	4,34	17,53	9,36	3,89
2,5	0,07	0,58	0,16	0,71	0,17	0,04	0,18	21,22	15,26	13,75	7,5	19,52	14,45	13,16
3	0,06	0,5	0,08	0,43	0,14	0,09	0,18	26,46	14,02	14,32	13,89	18,54	13,12	17,5
3,5	0,05	0,41	0,06	0,29	1,23	0,06	0,18	25,76	14,55	17,16	15,78	20,58	14,59	22,32
4	0,06	0,37	0,07	0,29	0,83	0,08	0,18	24,45	12,07	16,7	17,54	20,79	14,94	23,87
4,5	0,05	1,18	0,09	0,17	0,5	0,08	0,24	26,62	11,91	16,57	17,26	18,44	12,83	22,25
5	0,07	0,59	0,08	0,16	0,33	0,03	0,21	16,59	15,19	12,37	17,23	20,6	13,05	25,51
5,5	0,08	0,32	1,16	0,14	0,21	0,08	0,66	24,32	11,76	11,89	9,64	15,55	13,39	22,23
6	0,09	0,67	0,94	0,16	0,15	0,07	1,65	30,76	13,1	15,14	13,35	15,97	9,82	23,43
7	0,46	0,33	0,28	0,19	0,15	0,01	0,55	25,8	15,66	11,04	14,02	13,74	7,63	26,92
8	0,16	1,77	0,12	3,11	0,8	0,05	0,27	22,93	14,46	12,21	13,05	12,18	3,91	23,14
9	0,09	0,53	0,09	0,73	0,34	0,05	0,17	25,82	15,28	12,4	12,25	9,44	0,17	25,01
10	1,86	0,69	0,07	0,15	0,18	1,32	0,26	16,18	14,05	15,89	9,87	0,92	2,29	23,27
11	0,28	0,69	0,03	0,13	0,19	0,11	0,24	15,08	12,74	14,97	7,7	0,92	2,37	17,68
12	0,28	0,51	0,03	0,16	2,13	0,08	0,22	2,02	7,38	8,17	3,9	0,59	1,06	1,69
14	0,22	0,23	0,02	0,12	0,21	0,07	0,23	2,13	0,56	0,44	3,94	0,26	1,13	1,51
18	0,09	2,55	0,04	0,22	0,65	0,09	0,2	1,28	0,51	0,21	2,44	0,22	1,09	0,95
22	0,19	0,29	0,07	0,15	0,19	0,24	0,17	0,44	0,37	0,14	0,39	0,08	0,52	0,36
26	0,19	0,66	0,07	0,18	0,44	0,16	2,24	7,19	0,33	0,1	20,28	0,06	4,73	0,2

Non-breeding season FSH (ng mL^{-1})

Time(hours)	Vehicle							C6						
-1	0,39	0,36	0,18	0,75	0,43	0,35	0,84	0,27	0,25	0,65	0,32	0,62	0,84	0,5
-0,5	0,36	0,34	0,16	0,76	0,42	0,47	0,9	0,34	0,25	0,62	0,3	0,55	0,82	0,52
0	0,4	0,42	0,22	0,56	0,51	0,4	0,98	0,38	0,25	0,73	0,33	0,51	0,73	0,42
0,5	0,37	0,47	0,24	0,59	0,46	0,49	0,97	0,41	0,24	0,98	0,59	0,61	0,92	0,54
1	0,39	0,31	0,21	0,52	0,46	0,38	0,94	0,71	0,31	1,11	0,51	0,77	0,89	0,47
1,5	0,3	0,38	0,26	0,51	0,45	0,4	0,94	0,71	0,42	1,15	0,67	0,66	0,91	0,58
2	0,36	0,38	0,31	0,46	0,47	0,46	0,96	0,97	0,48	1,5	0,78	0,88	1,01	0,71
2,5	0,38	0,45	0,24	0,52	0,51	0,44	0,96	1,44	0,49	1,87	1,11	1,19	1,1	0,98
3	0,3	0,4	0,28	0,61	0,58	0,49	0,95	2,04	0,87	1,99	1,41	1,44	1,2	1,38
3,5	0,37	0,38	0,31	0,45	0,52	0,51	1	2,27	0,89	2,15	1,43	1,71	1,33	2,15
4	0,3	0,36	0,27	0,44	0,6	0,52	1,03	2,52	1,02	2,04	1,54	1,75	1,38	3,53
4,5	0,33	0,44	0,29	0,38	0,72	0,43	1,17	2,34	1,01	1,98	1,65	2,14	1,2	3,4
5	0,27	0,44	0,3	0,45	0,76	0,38	1,08	2,22	1	1,95	1,76	1,93	1,23	4,29
5,5	0,32	0,36	0,43	0,39	0,69	0,57	1,12	2,31	0,97	1,69	1,52	2,23	1,18	3,88
6	0,29	0,4	0,41	0,43	0,55	0,41	1,03	2,25	1,19	1,64	1,43	2,21	1,22	3,88
7	0,28	0,24	0,34	0,47	0,58	0,37	0,94	2,13	1,01	1,43	1,32	2,01	1,07	3,59
8	0,31	0,27	0,36	0,37	0,57	0,44	0,97	1,95	1,02	1,5	1,36	1,79	0,97	3,4
9	0,31	0,28	0,28	0,5	0,45	0,48	0,89	1,8	1,14	1,48	1,14	1,74	0,8	2,74
10	0,45	0,36	0,21	1,07	0,59	0,61	0,88	1,53	1,4	1,61	1,03	1,51	0,67	2,27
11	0,34	0,3	0,28	0,78	0,64	0,45	0,84	1,26	1,3	1,82	0,87	1,29	0,51	1,65
12	0,32	0,45	0,24	0,89	0,83	0,41	0,99	0,47	1,29	1,21	0,56	0,43	0,34	0,68
14	0,35	0,5	0,25	0,91	0,84	0,44	0,99	0,46	1,36	1,34	0,57	0,42	0,33	0,62
18	0,38	0,44	0,29	1	0,93	0,43	0,92	0,39	0,84	0,66	0,45	0,38	0,34	0,71
22	0,64	0,19	0,26	0,72	0,69	0,37	0,82	0,17	0,29	0,51	0,19	0,32	0,2	0,83
26	0,74	0,35	0,26	0,72	0,88	0,22	0,75	0,37	0,3	0,92	1,52	0,32	0,38	0,56

Non-breeding season progesterone (ng mL^{-1})

Days after injection	Vehicle						C6					
1	0	0,4	0	0	0,5	0	0	0	0	0	0	0
2	1	0,6	0,5	0,4	0	0	1,4	0,5	0	0,8	0,6	0,8
3	0,4	1,7	0	1,2	0,6	0,4	1,3	1,2	1,4	1,8	1,5	0,5
4	0,9	1,1	0	0	0,5	0,3	1,9	1,9	1,7	2,6	1,6	1,7
5	0,9	0,7	0,3	0	0	0,7	1,2	3	2,8	3,3	2,2	3,6
6	0,9	0,5	0,5	0,4	0	0	3,7	3,9	4,8	5,4	3	3,4
7	0,3	0,6	0	0,4	0,7	0,7	4,4	5,6	6	5,8	4,8	4,9

Onset of the breeding season LH (ng mL^{-1})

Time (hours)	Vehicle					C6				
-1	0,3	1,1	0,5	0,3	1,1	0,2	1	0,2	0,2	0,4
-0,5	0,2	0,7	0,2	0,2	0,6	0,7	0,7	0,2	0,1	0,7
0	0,2	0,4	0,2	0,9	0,4	0,2	0,4	0,1	0,2	0,4
0,5	0,2	0,5	0,4	0,5	1,1	0,2	1,1	1,2	1,3	0,6
1	0,2	1	0,4	1,9	0,7	0,4	1,9	2,1	1,3	1,6
1,5	0,2	0,7	0,3	0,2	0,3	1,7	5	3,3	3,1	2,3
2	0,2	0,5	0,9	0,2	0,3	2,1	10,5	5,3	6,8	3,7
2,5	2,8	0,7	0,6	1,2	1	3,2	34	9,5	24,2	6,3
3	1,4	1,7	0,5	0,6	0,7	4,3	67,8	15,3	35,1	19,7
3,5	0,7	0,9	1,3	0,3	0,4	4,8	52,5	25,4	53,1	29,8
4	0,5	0,6	0,8	0,2	0,4	6,9	152,1	30,7	84,4	37,8
4,5	0,4	0,8	0,6	0,2	1	9,1	85,4	36,4	71,5	40
5	0,3	1,5	1,3	0,2	0,7	8,1	67,7	29,4	73,1	37
5,5	0,3	0,8	0,8	1	0,4	9,9	69,8	45,9	57,3	50,6
6	0,2	0,6	0,5	0,5	0,3	11,7	66,7	42,9	66,5	57,4
7	0,3	1,3	0,6	0,2	0,8	10,8	29,6	29,9	50,9	59,6
8	0,2	0,6	0,3	0,2	0,3	7,9	20,4	27,8	57,7	24,5
9	0,3	1,2	0,4	0,7	0,2	17,1	7,2	26,3	42,5	7,1
10	0,2	0,6	0,2	0,2	0,2	6,5	4,8	22,4	48,7	6
11	0,7	1,1	0,3	0,2	0,5	4,9	2,6	23	16,6	3,1
14	1,6	0,7	0,3	0,2	0,5	1,7	1	7,7	2,8	1
18	0,2	0,5	0,4	0,8	0,3	1,6	0,5	2,4	1,2	0,7
22	1,3	1	2,1	1,7	0,7	0,9	0,3	0,6	0,5	0,3
26	1,9	1,4	126,6	0,2	67,6	0,3	0,2	0,2	0,5	0,3

Onset of the breeding season FSH (ng mL^{-1})

Time (hours)	Vehicle					C6				
-1	0,71	0,72	0,34	0,35	0,15	0,27	0,68	0,15	0,9	0,39
-0,5	0,71	0,75	0,28	0,37	0	0,25	0,58	0,15	0,94	0,37
0	0,66	0,76	0,22	0,35	0,11	0,22	0,72	0,14	0,93	0,4
0,5	0,65	0,68	0,3	0,38	0,12	0,35	0,68	0,18	1,07	0,4
1	0,63	1,1	0,32	0,33	0,12	0,26	0,77	0,29	1,14	0,47
1,5	0,62	0,62	0,31	0,25	0,17	0,39	0,98	0,57	1,27	0,4
2	0,57	0,47	0,26	0,25	0,21	0,59	0,96	1	1,26	0,6
2,5	0,83	0,56	0,37	0,34	0,2	0,81	1,05	1,53	1,36	0,99
3	0,67	0,54	0,4	0,41	0,25	1,13	1,17	2,23	1,55	1,46
3,5	0,64	0,39	0,34	0,3	0,3	1,89	1,19	3,08	1,67	1,77
4	0,61	0,39	0,33	0,31	0,33	2,2	1,25	2,8	1,71	2,25
4,5	0,36	0,46	0,41	0,38	0,36	2,54	1,32	3,22	1,85	2,54
5	0,65	0,36	0,4	0,37	0,33	2,56	1,29	2,98	1,77	2,69
5,5	0,82	0,34	0,4	0,38	0,32	3,13	1,35	2,5	1,69	2,44
6	0,65	0,32	0,37	0,38	0,3	3,41	1,32	2,49	1,64	2,74
7	0,6	0,35	0,47	0,45	0,27	3,14	1,27	1,91	1,55	1,82
8	0,56	0,31	0,44	0,36	0,22	3,09	1,2	1,38	1,57	1,87
9	0,55	0,52	0,47	0,39	0,18	2,56	1,28	1,01	1,52	1,44
10	0,56	0,4	0,51	0,44	0,14	2,03	0,95	0,82	1,4	1,16
11	0,58	0,42	0,48	0,35	0,15	1,32	0,73	0,31	1,41	0,8
14	0,81	0,51	0,45	0,54	0,16	0,57	0,54	0,39	1,06	0,5
18	0,54	0,47	0,5	0,53	0,25	0,54	0,42	0,64	0,57	0,49
22	0,5	0,68	0,51	0,37	0,25	1,38	0,87	1,05	0,37	0,78
26	0,48	0,4	0,64	2,05	1,99	1,57	1,35	1,03	0,33	1,08

Onset of the breeding season progesterone (ng mL^{-1})

Days after injection	Vehicle					C6				
1	0	0	0	0,5	0	0	0	0	0	0
2	0	0	0	0,4	0	0	0	0	0,5	0,3
3	0,4	0,4	0,3	0,4	0,5	0,9	1,5	1,3	1	1,7
4	0,4	2,1	0	0,7	2,7	2,6	3,8	3,7	1,6	5,1
5	0	4,3	0	1,2	4,9	4,2	6,3	4,9	2,5	7,6
6	0	6,4	0	2,7	7,2	6,1	8,1	8,2	4,2	9,5
7	0	8,1	0,4	4,7	9,5	7,3	10,1	7,6	4,1	11
